# Genome Sequence and Annotation of a Human Infection Isolate of Escherichia coli O26:H11 Involved in a Raw Milk Cheese Outbreak

**DOI:** 10.1128/genomeA.01568-14

**Published:** 2015-02-19

**Authors:** Wessam Galia, Patricia Mariani-Kurkdjian, Estelle Loukiadis, Stéphanie Blanquet-Diot, Françoise Leriche, Hubert Brugère, Ayaka Shima, Eric Oswald, Benoit Cournoyer, Delphine Thevenot-Sergentet

**Affiliations:** a Université de Lyon, Research Group on Bacterial Opportunistic Pathogens and Environment, UMR 5557 Ecologie Microbienne, CNRS, VetAgro Sup, and Université Lyon 1, Lyon, France; b Université de Lyon, VetAgro Sup, Campus Vétérinaire de Lyon, Laboratoire d’Étude des Microorganismes Alimentaires Pathogenes, French National Reference Laboratory for *Escherichia coli* including Shiga Toxin-Producing *E. coli*, Marcy l’Etoile, France; c Université de Clermont-Ferrand, VetAgro Sup, Campus Agronomique de Lempdes, Unité CALYTISS, Lempdes, France; d Clermont Université, Université d'Auvergne, Centre de Recherche en Nutrition Humaine Auvergne, EA 4678 CIDAM, BP 10448, Clermont-Ferrand, France; e INRA, USC1360, Toulouse, France; f INSERM UMR1043/CNRS UMR5282/Université Toulouse III Centre de Physiopathologie de Toulouse Purpan (CPTP), Toulouse, France; g Laboratory associated with the National Reference Centre for *Escherichia coli* and *Shigella*, Robert Debré Hospital, Paris, France

## Abstract

The consumption of raw milk cheese can expose populations to Shiga toxin-producing Escherichia coli (STEC). We report here the genome sequence of an E. coli O26:H11 strain isolated from humans during the first raw milk cheese outbreak described in France (2005).

## GENOME ANNOUNCEMENT

Enterohemorrhagic Escherichia coli can cause foodborne diseases ranging from simple diarrhea to hemorrhagic colitis and life-threatening complications, such as hemolytic-uremic syndrome. E. coli O157:H7 is implicated in the majority of outbreaks and hemolytic-uremic syndrome (HUS) cases (1). However, other serotypes, such as E. coli O26:H11, have also been implicated in outbreaks (2, 3). Yet, little is known about the genomic diversity that exists among E. coli O26:H11 pathogen populations or how various genotypes of this pathogen relate to the development and severity of the clinical manifestations in infected patients. The genome sequence data bring more clarity to both the evolutionary processes and virulence factors of this important pathogen.

Genomic DNA was extracted from an overnight culture using the DNeasy blood and tissue kit (Qiagen). A sequencing library was prepared with Nextera XT version 3 chemistry (Illumina). Following fragmentation, end reparation, and sample tagging, the Illumina MiSeq platform sequencer produced 300-bp paired-end reads that were obtained from 700-bp inserts, yielding an average coverage of 1,000×. The program FastQC version 0.10.1 (http://www.bioinformatics.babraham.ac.uk/projects/fastqc/) was applied to check the quality of the generated 16,638,445 reads. This data set was employed for the *de novo* sequence assembly by various assemblers, including SPAdes version 3.0.0 (4), Minia version 1.6088 (5, 6), Velvet version 1.2.10 (7), and MaZuRCA version 2.2.1 (8). The sequence contigs obtained from the assemblers were subsequently input into the Contig Integrator for Sequence Assembly (CISA) to generate an integrated set of contigs (9). Compared with the assemblies generated by each assembler, a hybrid set of assemblies of superior contiguity and accuracy was obtained by CISA. Next, 102 hybrid contigs were given, resulting in a total genome size of 5,686,107 bp and an *N*_50_ of 107,803 bp. The resulting contigs were ordered by alignment to the reference genome of E. coli O26:H11 strain 11368 (10) using Mauve Contig Mover version 2.3.1 (11, 12). Functional annotation was carried out using the tools of the MicroScope platform (13), and the annotated genome was implemented in the public EscherichiaScope database (see https://www.genoscope.cns.fr/agc/microscope/home/index.php). The assembly contains 5,781 genomic objects, among which are 5,611 coding sequences, 10 rRNA genes, 92 tRNA genes, and 68 noncoding RNAs. The genome G+C contents were relatively close to those of the reference genomes (50.6%).

A detailed report of the phylogenetic analysis of this draft sequence will be included in a future publication.

### Nucleotide sequence accession numbers.

The draft genome sequence of the E. coli O26:H11 strain 21765 in this study has been deposited as a whole-genome shotgun project at the European Nucleotide Archive (http://www.ebi.ac.uk/ena/data/view) under the contig accession numbers CDLB01000001 to CDLB01000102.
