# Correction for Galia et al., Genome Sequence and Annotation of a Human Infection Isolate of *Escherichia coli* O26:H11 Involved in a Raw Milk Cheese Outbreak

**DOI:** 10.1128/genomeA.00880-15

**Published:** 2015-07-30

**Authors:** Wessam Galia, Patricia Mariani-Kurkdjian, Sylvere Bastien, Estelle Loukiadis, Stéphanie Blanquet-Diot, Françoise Leriche, Hubert Brugère, Ayaka Shima, Eric Oswald, Benoit Cournoyer, Delphine Thevenot-Sergentet

**Affiliations:** a Université de Lyon, Research Group on Bacterial Opportunistic Pathogens and Environment, UMR 5557 Ecologie Microbienne, CNRS, VetAgro Sup, and Université Lyon 1, Lyon, France; b Université de Lyon, VetAgro Sup, Campus Vétérinaire de Lyon, Laboratoire d’Étude des Microorganismes Alimentaires Pathogenes, French National Reference Laboratory for *Escherichia coli* including Shiga Toxin-Producing *E. coli*, Marcy l’Etoile, France; c Université de Clermont-Ferrand, VetAgro Sup, Campus Agronomique de Lempdes, Unité CALYTISS, Lempdes, France; d Clermont Université, Université d’Auvergne, Centre de Recherche en Nutrition Humaine Auvergne, EA 4678 CIDAM, BP 10448, Clermont-Ferrand, France; e INRA, USC1360, Toulouse, France; f INSERM UMR1043/CNRS UMR5282/Université Toulouse III, Centre de Physiopathologie de Toulouse Purpan (CPTP), Toulouse, France; g Laboratory associated with the National Reference Centre for *Escherichia coli* and *Shigella*, Robert Debré Hospital, Paris, France; h PRABI and Université Lyon 1, Lyon, France

## AUTHOR CORRECTION

Volume 3, no. 1, e01568-14, 2015. Page 1: The byline and affiliation line should read as given above.

